# Protein Quantification and Imaging by Surface‐Enhanced Raman Spectroscopy and Similarity Analysis

**DOI:** 10.1002/advs.201903638

**Published:** 2020-04-16

**Authors:** Hyunku Shin, Seunghyun Oh, Daehyeon Kang, Yeonho Choi

**Affiliations:** ^1^ Department of Bio‐convergence Engineering Korea University Seoul 02841 Republic of Korea; ^2^ School of Biomedical Engineering Korea University Seoul 02841 Republic of Korea; ^3^ Department of Bioengineering Korea University Seoul 02841 Republic of Korea

**Keywords:** plasmonics, protein imaging, protein quantification, similarity analysis, surface‐enhanced Raman spectroscopy

## Abstract

Protein quantification techniques such as immunoassays have been improved considerably, but they have several limitations, including time‐consuming procedures, low sensitivity, and extrinsic detection. Because direct surface‐enhanced Raman spectroscopy (SERS) can detect intrinsic signals of proteins, it can be used as an effective detection method. However, owing to the complexity and reliability of SERS signals, SERS is rarely adopted for quantification without a purified target protein. This study reports an efficient and effective direct SERS‐based immunoassay (SERSIA) technique for protein quantification and imaging. SERSIA relies on the uniform coating of gold nanoparticles (GNPs) on a target‐protein‐immobilized substrate by simple centrifugation. As centrifugation induces close contact between the GNPs and target proteins, the intrinsic signals of the target protein can be detected. For quantification, the protein levels in a cell lysate are estimated using similarity analysis between antibody‐only and protein‐conjugated samples. This method reliably estimates the protein level at a sub‐picomolar detection limit. Furthermore, this method enables quantitative imaging of immobilized protein at a micrometer range. Because this technique is fast, sensitive, and requires only one type of antibody, this approach can be a useful method to detect proteins in biological samples.

## Graphical abstract

An immunoassay technique based on direct surface‐enhanced Raman spectroscopy and similarity analysis is demonstrated in this study. The proposed method relies on the sedimentation of plasmonic nanoparticles on a protein conjugated substrate, providing sub‐picomolar quantification and reliable imaging of proteins.

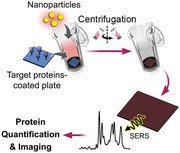

## 1 Introduction

Protein detection is of considerable interest in biomedical engineering because proteins play vital roles as byproducts and mediators of various biochemical activities.^[^ ^1^ ^]^ Thus, proteins have emerged as effective biomarkers for indicating biofunctional statuses related to genomic misregulation, metabolic processes, and disease progression.^[^ ^2^ ^]^ Because protein biomarkers can indicate the types and progression of diseases, the detection of proteins in body fluids is important for liquid biopsy of diseases.^[^ ^3^ ^]^ Accordingly, many protein assay techniques, such as the enzyme‐linked immunosorbent assay (ELISA), fluorescence, and localized surface plasmon resonance‐based detection techniques, have long led significant advances in protein detection.

Recently, spectroscopic methods such as Raman spectroscopy are in great interest as promising techniques for detecting proteins. Owing to the advantage of offering molecular vibrational and rotational information, Raman spectroscopy can provide insights associated with the structural properties of proteins as compared to other extrinsic analytical methods.^[^ ^4^ ^]^ However, weak scattering intensity of Raman scattering is a major obstacle to make difficulty in detecting proteins sensitively. For the reason, surface‐enhanced Raman spectroscopy (SERS) has been introduced. SERS can enhance the subtle Raman scattering signal by an electromagnetic field in a nanogap between metallic nanostructures.^[^ ^5^ ^]^ Among the SERS approaches, direct SERS is the simplest because it detects the intrinsic Raman signal of analytes without indispensable tagging procedures.^[^ ^6^ ^]^ Moreover, the direct SERS is widely known to be an ultrasensitive method capable of femtomolar detection.^[^ ^7^ ^]^

However, direct SERS has major limitations regarding application to biological samples: 1) Biological samples contain numerous substances that share Raman bands with the target protein. These molecules can produce a false‐positive signal and interfere with the detection of the target protein signal. The target protein can be purified and isolated from the sample, but the protein purification requires a complicated process and a large amount of the sample. Therefore, an immunological technique for capturing target proteins selectively with antibodies can be applied.^[^ ^8^ ^]^ However, antibodies that adhere to the SERS substrate can be physical obstacles and may extend the distance from the SERS effective area to the target protein and become interfering substances, thereby diminishing the desired signal.^[^ ^9^ ^]^ For this reason, antibodies have been used restrictively in direct SERS detection. Therefore, locating the target protein close to the SERS probe is essential, to minimize the interference by antibodies. 2) The spectral features of proteins are usually complex and heterogeneous for each measurement. Different measurement setups and preprocessing methods among research groups can be linked to a disaccord of intensity and spectral shape. Moreover, because several factors such as interfacial properties of nanoparticles, structural flexibility of proteins, and different adsorption of proteins reduce reproducibility, they may make the analysis of complex spectral features more difficult.^[^ ^10^ ^]^

**Figure 1:**
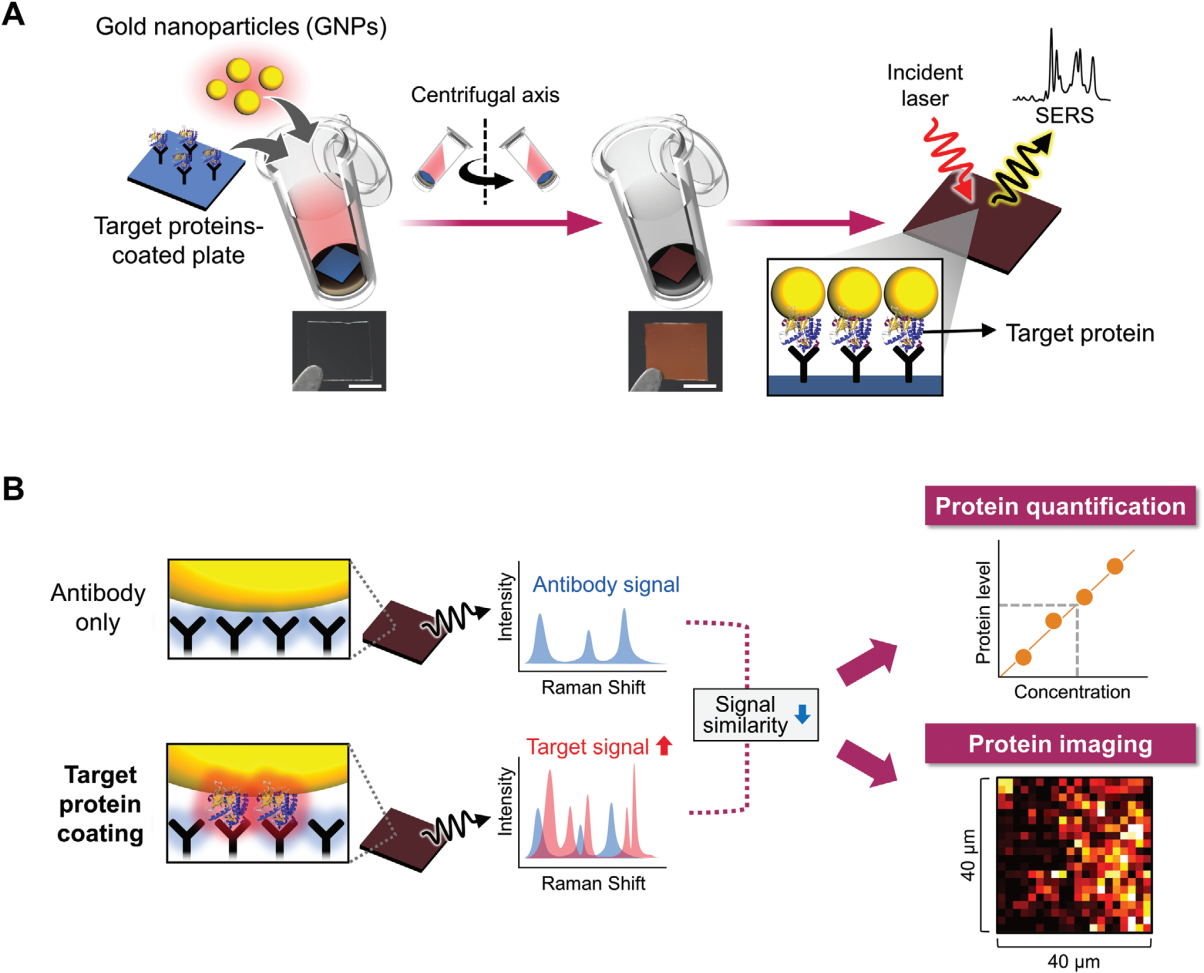
Protein quantification and imaging using the SERSIA. A) Schematic overview of the procedure for GNP coating on a target protein‐immobilized substrate by centrifugation, and the SERS characterization of the protein. The photographs represent the substrate before and after GNP coating. The scale bar is 2.5 mm. B) Protein quantification and imaging by combining SERS and similarity analysis.

Herein, we present a direct SERS‐based immunoassay (SERSIA) that combines the SERS technique and similarity analysis. Our method can overcome the challenges of immunological application and heterogeneity in signal patterns. Our method is based on the close contact between gold nanoparticles (GNPs) and the target protein that is selectively immobilized on an antibody‐coated substrate (**Figure** **1**A). We simply and rapidly induce GNPs coating by centrifugation, without any functionalization on the GNP surface. Then, repetitive centrifugation cycles promote the GNP coating and generate an effective SERS hot spot easily. Because the GNPs closely contact the target proteins, they exhibit the desired signal effectively, while minimizing the influence of the antibody. Moreover, we identify the protein level through a similarity analysis of the spectra between antibody‐only and target‐protein‐immobilized samples (Figure 1B). As immobilized target proteins increase in number, the proteins yield their intrinsic signal and the similarity to the antibody‐only sample diminishes. Therefore, we estimate the immobilized protein level based on the mathematical similarity. This approach does not consider only a specific Raman band and thus overcomes the complexity and heterogeneity issues of the spectral features. In addition, by scanning the spectra along the surface, we can image the distribution of immobilized proteins.

## 2 Results and Discussion

### 2.1 GNP Coating by Centrifugation

**Figure 2:**
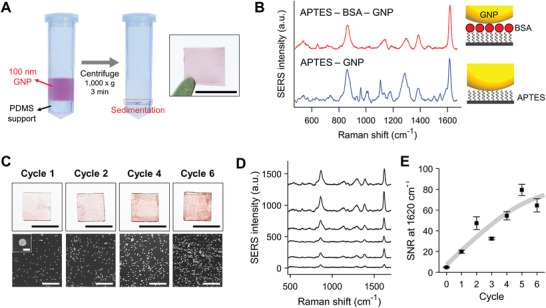
GNP coating by centrifugation for detecting intrinsic SERS signal of proteins. A) GNP coating through centrifugation of GNPs on a protein‐immobilized substrate. The inset image shows the GNP‐coated cover glass substrate after centrifugation. The scale bar is 5 mm. B) SERS signals of APTES‐GNP and APTES‐BSA‐GNP. C) Photographs and SEM images of GNP coating with repeated centrifugation cycles. The scale bars represent 5 mm (photograph), 2 µm (SEM), and 100 nm (SEM inset). D) SERS signal of the BSA‐conjugated sample, and E) SNR in the SERS signal at 1620 cm^−1^ with repeated cycles.

We attempted to settle GNPs at a condition of 1000 × *g*, which can be easily performed using common centrifuge devices. First, 30, 50, and 100 nm GNP colloidal solutions were centrifuged to determine the size of nanoparticles that can be precipitated by the condition. After centrifugation for 3 min, sedimentation of 30 and 50 nm GNPs was not significant and most particles remained at the supernatant (Figure S1, Supporting Information). By contrast, despite the short centrifugation time, the 100 nm GNPs were well‐precipitated, and the supernatant changed color from red to transparent (**Figure** **2**A). To test the ability of GNPs coating by centrifugation, we first coated GNPs onto a 3‐aminopropyltriethoxysilane (APTES)‐functionalized cover glass substrate. The glass substrate was cut into square shapes having sides of 5 mm, for placement in a 2 mL centrifuge tube. To prevent the substrate from being damaged by the curved bottom of the tube, we used a polydimethylsiloxane (PDMS) support with a flat top surface. The cover glass was located on the PDMS support, and the 100 nm GNP colloidal solution was poured into the tube. After centrifugation, the resulting glass showed a noticeable red color, indicating that the GNPs were coated on the surface. The coated GNPs were also observed using scanning electron microscope (SEM; Figure S2, Supporting Information).

To characterize the SERS substrate, we attached 4‐aminothiophenol (4‐ATP) to the surface of the GNPs and then the enhancement effect and signal uniformity were evaluated. The enhancement factor (EF) of the substrate was calculated to be 2.0 × 10^7^ (see Supporting Information). Thus, our substrate yielded comparable enhancement effect to aggregate nanoparticles without preconcentration or cumbersome chemical treatment.^[^ ^11^ ^]^ Moreover, because the plasmonic nanoparticles as SERS probes were coated on the entire substrate, SERS signals could be detected from anywhere. To evaluate the uniformity of SERS detection, the SERS spectra at 2 µm intervals and random locations were observed (Figure S3, Supporting Information). As a result, the substrate exhibited a constant signal intensity at the characteristic Raman bands of 4‐ATP, suggesting the excellent signal uniformity of our SERS substrate.

### 2.2 SERS Detection of Proteins by Centrifugation‐Induced GNP Coating

Our approach to detect the intrinsic SERS signal of target proteins was to locate GNPs close to the protein conjugated on the surface of the substrate. Therefore, the GNPs must be attached to proteins coated on the substrate, without additional linkers such as primary amine or thiol groups. As a proof of concept, we performed the same coating procedure on substrates in which bovine serum albumin (BSA) was bound on the surface. Before the SERS measurements, we examined the signal stability during our measurement, as excessive irradiation from a laser can lead to fluctuations in signals due to a burning event. During 10 s of exposure time, the fluctuations in the signals were within the natural noise range (Figure S4, Supporting Information). Also, because Raman bands assigned to amorphous carbon did not appear, we confirmed that there was no burning event.^[^ ^12^ ^]^

Both samples exhibited strong SERS bands at 861, 1286, and 1600 cm^−1^ assigned to citrate covering the nanoparticle surface (Figure S5, Supporting Information). Notably, the APTES‐BSA‐GNP sample exhibited a different signal pattern from the APTES‐GNP sample (Figure 2B). The APTES‐GNP sample produced stronger intensities at several bands. Among the bands, the stronger SERS intensity at 539, 608, 962, 1013, 1133, and 1502 cm^−1^ bands correlated to the reported characteristic SERS peaks originating from the self‐assembled monolayer of APTES.^[^ ^13^ ^]^ Interestingly, these bands of APTES vanished in the APTES‐BSA‐GNP sample, whereas several bands maintained their intensity at 644, 1029, and 1454 cm^−1^ that are assigned to tyrosine, phenylalanine, and CH_2_ bending, respectively.^[^ ^14^ ^]^ We assumed that the BSA molecules played a role in extending the distance between GNP and APTES and placing the APTES far from the SERS effective area.

### 2.3 Additional Enhancement in SERS Signal by Repeated GNP Coating

Because enhancement of intensity is one of the major interests in direct SERS, we investigated an easy and simple method of amplifying the intensity using our method. For this reason, repetitive centrifugation was performed to coat more GNPs on the protein‐conjugated substrate. At the intervals between cycles, only a process to remove the supernatant and add 500 µL of colloidal solution was performed, without an additional washing process. As a result, the repetitive centrifugation promoted the coating of GNPs and led to greater signal enhancement. With an increase in the number of cycles, more GNPs were transferred onto the substrate, and the GNPs showed a darker burgundy color (Figure 2C). SEM images indicated that additional GNPs were covered on the substrate with the increasing number of centrifugation cycles. As the number of cycles increased, the GNP‐coated area in the SEM image was elevated. Accordingly, the SERS signal intensity was gradually enhanced (Figure 2D). We evaluated the signal‐to‐noise ratio (SNR) at 1620 cm^−1^ assigned to the amide acid ring and NH_2_. After six cycles, the SNR increased 14‐fold compared with that of one cycle (Figure 2E). This additional sedimentation could also be conducted with other plasmonic nanoparticles (Figure S6, Supporting Information). Silver nanoparticles and gold nanorods (GNRs) with citrate were sufficiently coated on the substrate via the repeated coating, offering viable options to select nanoparticles for SERS considering cost and optical setup. However, coatings of GNRs with cetrimonium bromide (CTAB) were negligible. Because CTAB‐capped nanoparticles are positively charged, the particle coating in our method could be associated with the interaction between several positively charged side chains of the BSA surface and the negatively charged citrate‐capped nanoparticle surface.^[^ ^15^ ^]^

### 2.4 SERSIA of the Target Proteins

The purpose of our method was to detect a target protein in a complex biological sample. The easiest means of capturing a specific protein in a biological sample is through conducting antibodies. As previously described, we confirmed that GNPs could be coated on a protein‐conjugated substrate using the centrifugation method. Therefore, we employed the same method with the antibody‐only and antigen–antibody samples. To bind the antibody on an APTES‐functionalized substrate, we utilized the method of Vashist et al. (Figure S7, Supporting Information).^[^ ^16^ ^]^ Epidermal growth factor receptor (EGFR) was first chosen as the antigen. When the same volume of GNP colloidal solution was used, no difference was observed in the coverage of the GNP‐coated area between the antibody‐only and EGFR‐immobilized samples (Figure S8, Supporting Information).

To test the ability of our method to detect the target protein selectively, we compared the spectrum with those obtained from an off‐target sample (Figure S9, Supporting Information). To observe the tendency of the signal difference, we employed principal component analysis (PCA), which converts high‐dimensional data such as a spectrum to dimension‐reduced data, and displays the tendency of data patterns. The results revealed that the off‐target sample exhibited no difference with the antibody‐only sample on the PCA score plot, whereas the target protein‐immobilized sample showed a different distribution. Because a separated distribution in the PCA results indicated that the samples exhibited different tendencies in their spectral patterns, these results support the claim that our method selectively identifies signal differences in the presence of target antigens.

**Figure 3:**
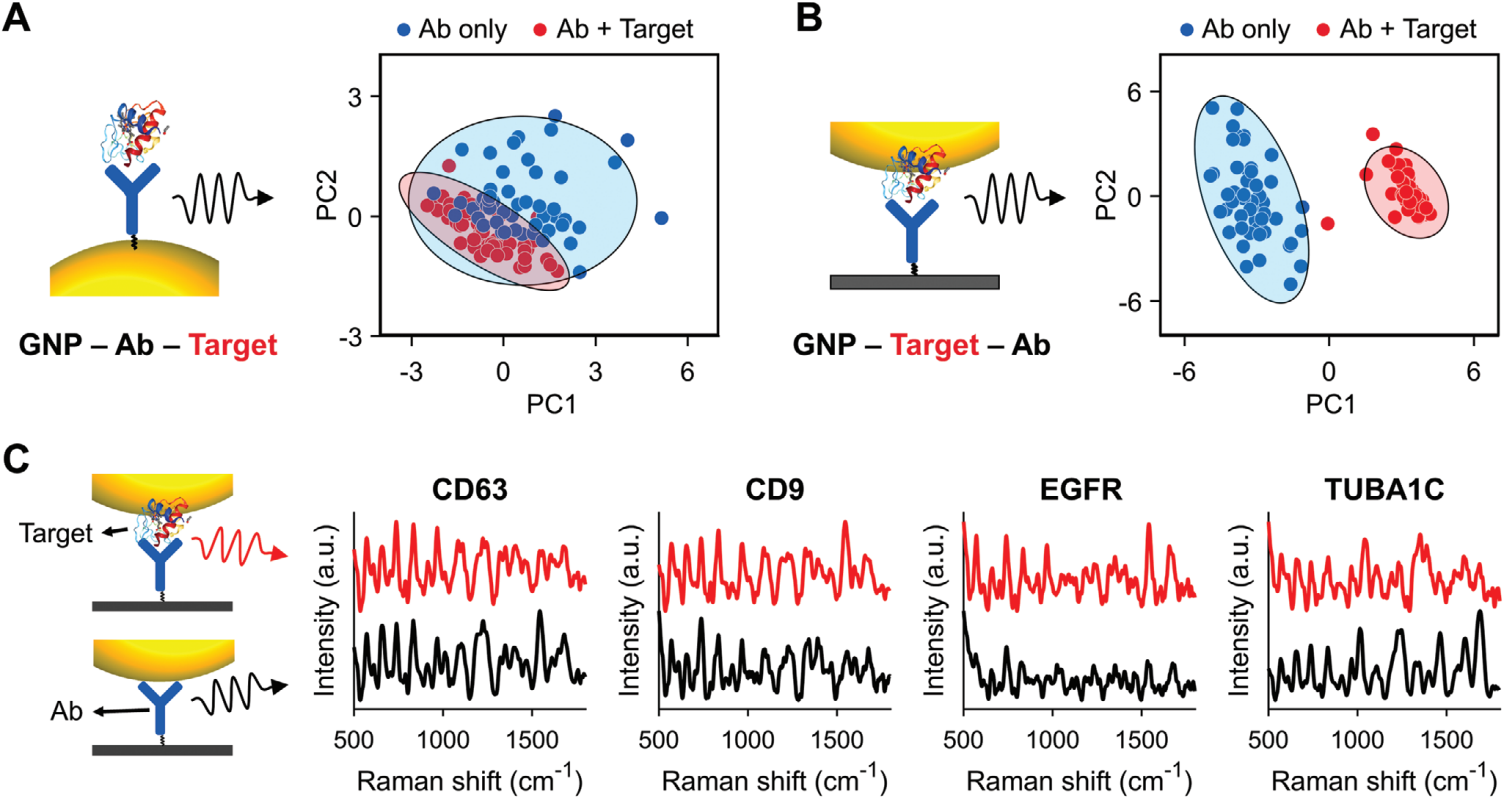
SERS detection of target proteins conjugated antibody substrate. A,B) Schematic and PCA score plots of A) the control case and B) our method case. C) SERS signals of protein‐conjugated samples and their corresponding antibody‐only samples. Each signal represents a mean of 25 spectra.

We further evaluated the effectiveness of our method for detecting the target protein by comparison with a control case in which an antibody was present between GNPs and the target protein. For the control case, GNPs were coated on an APTES‐functionalized substrate using the same centrifugation method. In addition, the anti‐EGFR antibodies were bound on the surfaces of GNPs, using the method of Braiek et al.^[^ ^17^ ^]^ The resulting antibody‐coated substrates were immersed in diluted H1666 cell lysate, which contained EGFR. PCA results against the SERS spectra of the samples showed that in the control case, the signals that appeared before and after EGFR immobilization were not separated, and their 95%‐confidence ellipses were overlapped (**Figure** **3**A). This means that their signal difference was minimal. By contrast, in our method, the signals following EGFR treatment were completely differentiated (Figure 3B). Because their 95%‐confidence ellipses were clearly distinguished, the difference was statistically significant. In the control case, the conjugated proteins may produce their intrinsic signal and generate a spectral change. However, this change may not be significant because the subtle signal of the proteins is merely added, whereas the signal of the antibody remains. By contrast, in our method, the conjugated proteins may not only reduce the antibody signal by extending the gap from SERS probes to the antibody but can also yield signals themselves. Accordingly, this simultaneous change in the signal can be attributed to the clear separation in the PCA result. Consequently, we confirmed that our SERSIA method is more effective at sensing the signal change after EGFR treatment.

We then applied our method for multi‐protein detection. For the purpose, we attempted to test our method using proteins that have importance in practical biological studies. Accordingly, the SERS signals of four proteins—CD63, CD9, EGFR, and TUBA1C—were observed in an A549 cell lysate (Figure 3C). Basically, CD9 and CD63 are known to be common exosome markers and have been widely used in extracellular vesicle researches.^[^ ^18^ ^]^ EGFR that belongs to a family of receptor proteins on the cell surface and has a vital role in the progression of lung cancer.^[^ ^3a^ ^]^ Tubulin alpha‐1C (TUBA1C) is a cytoskeleton‐related protein and reported to abundantly exist in tumor tissues.^[^ ^19^ ^]^ Also, because these proteins have no chromophores in their structure, identifying their Raman signal as compared with that proteins with chromophores is considerably more difficult. Because most proteins do not have chromophores, detecting such proteins for practical application to diverse proteins is critical. To capture the proteins, the glass substrates were coated with antibodies of the proteins. In SERS characterization, both samples exhibited discernable and various Raman peaks in the fingerprint regions of organic molecules. In particular, the characteristic peaks at ≈1243 cm^−1^ and ≈1673 cm^−1^, which were derived from the predominant *β*‐sheet structure in immunoglobulin G, were observed in all antibody samples.^[^ ^20^ ^]^ The target‐protein‐immobilized samples shared similar Raman peaks with the corresponding antibody samples, but they had different signal intensities at several peaks.

### 2.5 Protein Quantification by Similarity Analysis of the Entire Signal Pattern

**Figure 4:**
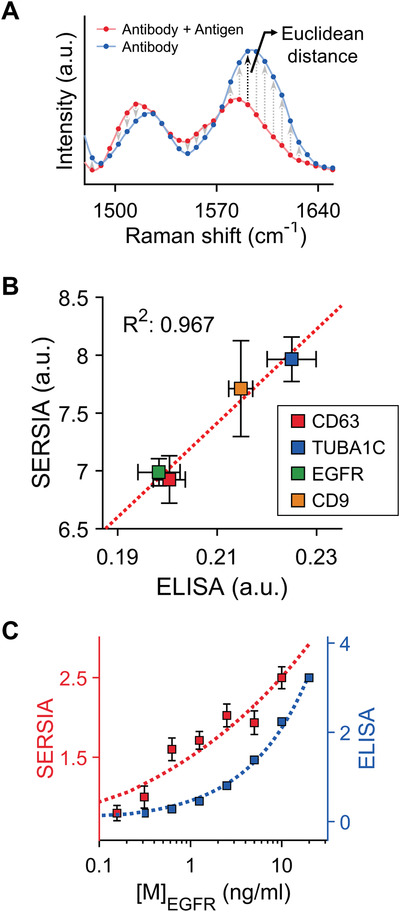
Protein quantification through SERSIA. A) Euclidean distance‐based similarity analysis for protein quantification. B) Correlation of the SERSIA and ELISA. *R* ^2^ is the coefficient of determination. C) Sensitivity comparison between the SERSIA and ELISA. The axes of the SERSIA and ELISA (B and C) represent the Euclidean distance and the absorbance at a wavelength of 450 nm, respectively.

We quantified the amount of target protein through signal similarity analysis between the antibody‐only and target‐protein‐immobilized samples. This similarity can be conceived in terms of distance.^[^ ^21^ ^]^ In particular, the Euclidean distance is one of the practical methods to evaluate the similarity. The distance can indicate the numerical similarity between the measured and reference spectral data (**Figure** **4**A). A shorter distance indicates greater similarity. For measured data *m* = (*m*_1_, *m*_2_,…, *m_n_*) and reference data *r* = (*r*_1_, *r*_2_,…, *r_n_*), the distance (*d*) between them can be expressed as follows

(1) $$d\left(m,r\right)=\sqrt{\sum_{i=1}^{n}{\left(m_{i}-r_{i}\right)}^{2}}$$

where *n* is the entire length of spectral data. An antigen‐conjugated sample may produce a different signal pattern from an antibody‐only sample, and the Euclidean distance in the signal pattern between both samples can indicate the protein level.

We further applied this approach to multi‐protein quantification in a cell lysate. The four proteins mentioned earlier were observed. Simultaneously, the indirect ELISA test was performed using the same cell lysate. To identify the correlation between our SERSIA and ELISA results, we compared the absorbance in ELISA and the similarity in SERSIA (Figure 4B). Interestingly, the protein levels estimated using the similarity were correlated with the ELISA result, with an *R* ^2^ value of 96.7%. This result suggested that the similarity‐based quantification of the SERSIA has a reliability comparable to the gold standard method for protein quantification.

To evaluate the sensitivity of the SERSIA, we compared the detection performance of SERSIA to that of sandwich ELISA. As the target protein, a lyophilized full‐length EGFR protein in the ELISA kit was used. First, we obtained SERS signals at linearly decreasing concentrations of EGFR in the range of 157 pg mL^−1^ to 2.5 ng mL^−1^. The protein level estimated using the SERSIA decreased as the concentration of treated proteins decreased (Figure 4C). To determine the significance of the ordered detection result, the Jonckheere trend test was performed. As a result, the *p*‐value was 6.0 × 10^−12^, thus the detection trend has an ordered trend by decreasing concentration. Moreover, the SERSIA could detect 157 pg mL^−1^ of EGFR. Because this concentration is 924 fm in the molarity unit, this result suggests that our method has the sub‐picomolar level of detection performance. The detection sensitivity of the SERSIA was comparable to that of the sandwich ELISA. However, the sandwich ELISA requires both capture and primary antibodies with different epitopes, whereas the SERSIA requires only one antibody to capture the target proteins.

### 2.6 Protein Imaging by Scanning SERS Signals

**Figure 5:**
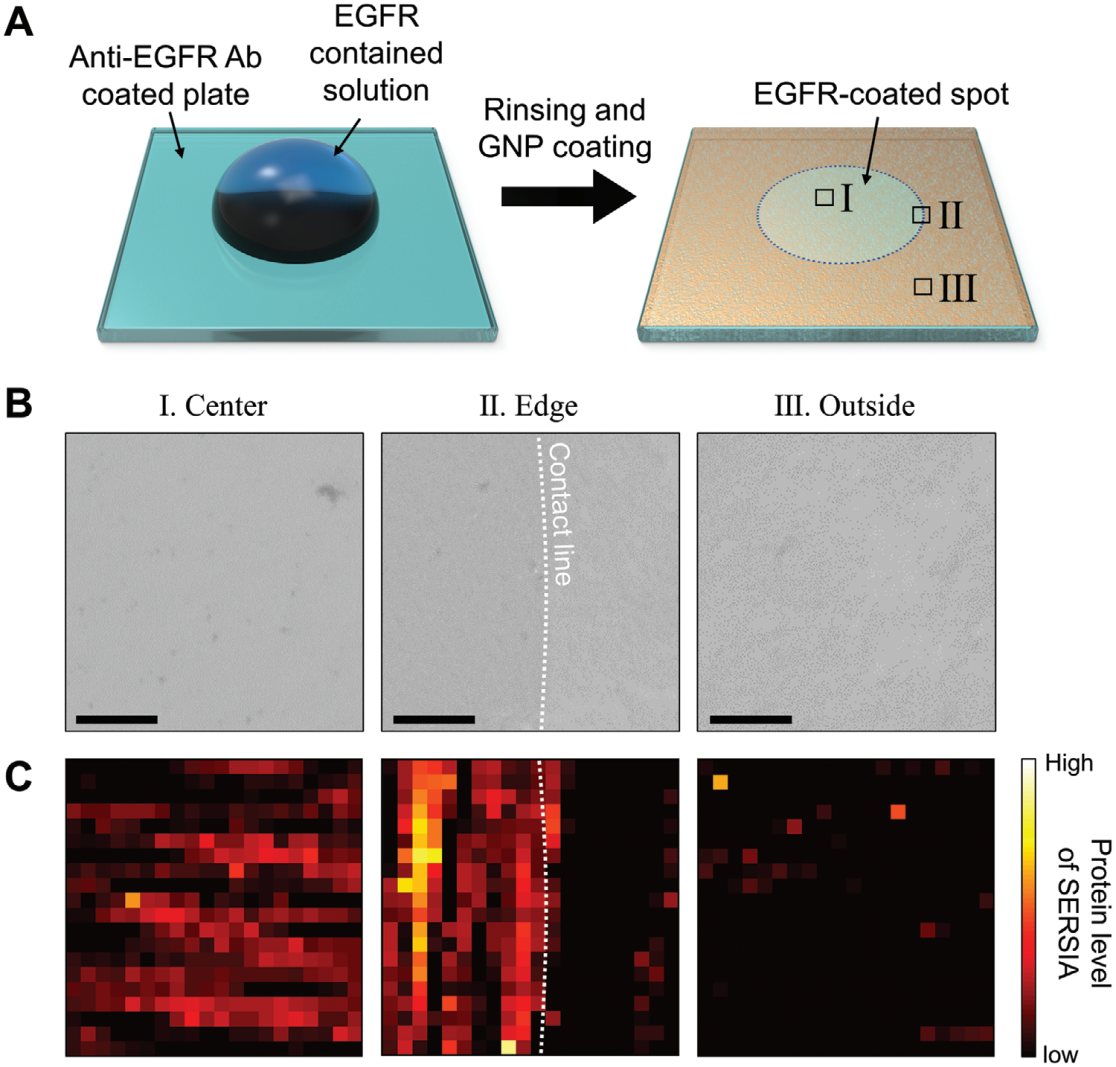
Protein imaging through SERSIA. A) Partial immobilization of proteins on the substrate. Soluble EGFR solution of 100 µg mL^−1^ was dropped onto the center of the substrate before GNP coating. B) Bright‐field microscopy images at (I) the center, (II) edge, and (III) outside of the protein‐immobilized spot. The scale bars indicate 10 µm. C) Protein imaging estimated by Euclidean distance. The size of the scanned image is 40 × 40 µm.

Our quantification method is based on signals obtained from a local point of SERS hot spots formed over a large area. This advantage offers feasibility for imaging protein concentrations in the local region through the similarity analysis. Therefore, as another application of our method, we applied the SERSIA to the imaging of the immobilized EGFR. To observe partially immobilized proteins at a liquid contact line, a small volume of the EGFR solution was dropped on the anti‐EGFR antibody‐coated substrate (**Figure** **5**A). Then, we monitored the protein level using the SERSIA at three spots—at the center, edge, and outside the dropped liquid. No significant difference was observed among the spots in the microscopic images of coated GNPs (Figure 5B). At each spot, 400 spectra were scanned by a shape of 20 × 20, at intervals of 2 µm. We then identified different protein levels among the spots. Most of the scanned spectra at the center, in which the target proteins abundantly existed, showed a high target‐protein level, whereas the spectra at the outside showed a relatively low protein level (Figure 5C). Interestingly, at the edge, partially immobilized proteins were observed. The majority of spectra at the inside of the liquid contact line produced a discernable protein level, whereas most outside spectra presented a low level. These results show that our method can monitor local protein immobilization.

We reported direct SERS‐based protein immunodetection combined with the similarity analysis of spectral data. Our method employs spectra obtained from local spots where proteins are immobilized. Because the detection area is small, the method may provide an opportunity to explore low volumes of analytes and monitor the protein–antibody binding dynamics. However, a future study is necessary to expand the types of proteins to which the method can be applied and to ensure the viability of the method. In addition, protein conformation such as isoforms and different orientations should be considered. Because various other techniques (e.g., machine learning) for analyzing multivariate data such as spectra are widely being introduced, we expect that the accuracy and reliability of our method can be improved by combining the techniques.

## 3 Conclusion

We demonstrated a SERS‐based immunoassay by centrifugation‐based GNP coating and similarity‐analysis‐based quantification. We coated GNPs onto a target protein‐coated antibody substrate through a simple centrifugation process and obtained the SERS signal of the target protein signal. Our method showed SERS signal amplification with repeated centrifugation steps. The proposed method exhibited advanced performance in the immunological approach of direct SERS. Thus, it allows substantially more effective detection of intrinsic signals from specific proteins in biological samples. Moreover, a Euclidean distance‐based similarity analysis was employed to identify the protein level. The detection sensitivity was comparable to that of the ELISA, and their correlation was well matched. In addition, this method proved the feasibility of protein imaging. We expect that our SERS approach can be utilized for protein quantification and imaging in various biological fields.

## 4 Experimental Section

##### Materials

HAuCl_4_, sodium citrate dihydrate, APTES, BSA, 3‐mercaptopropionic acid (MPA), *N*‐hydroxysuccinimide, penicillin, and streptomycin were purchased from Sigma‐Aldrich (St. Louis, MO). 1‐ethyl‐3‐(3‐(dimethylamino)propyl)carbodiimide (EDC) was purchased from Daejung Chemicals (KR). Anti‐CD9 (sc‐13118), anti‐CD63 (sc‐5275), and anti‐EGFR (sc‐373746) antibodies were purchased from Santa Cruz Biotechnology (Dallas, TX). RPMI 1640 and fetal bovine serum (FBS) were purchased from GE Healthcare (Chicago, IL). A cell lysis buffer (10×) and phenylmethylsulfonyl fluoride (PMSF) were purchased from Abcam (UK). EGFR lyophilized powder (E2645) for the off‐target test and imaging was purchased from Sigma‐Aldrich (St. Louis, MO). GNP colloidal solutions of one optical density were purchased from nanoComposix (San Diego, CA). Full‐length EGFR ELISA kit was purchased from Invitrogen (Carlsbad, CA)

##### Cell Culture and Lysis

Cell lines were maintained in RPMI 1640 supplemented with 10% FBS, penicillin (100 U mL^−1^), and streptomycin (100 mg mL^−1^), and were incubated at 37 °C in 5% CO_2_. The FBS was obtained from supernatants through ultracentrifugation overnight at 4 °C. All cells were grown to 50% confluency and incubated for 48 h. Each supernatant was collected after 48 h. The cells in the flask were harvested using a cell scraper with cold (4 °C) PBS. The PBS, including the scraped cells, was centrifuged in 500× *g*, and the pellet was stored at −80 °C until the downstream experiment was conducted. For lysis, PMSF (1 mm) and 50 µL of 1× diluted cell lysis buffer were mixed and added to the pellet in a 1.5 mL tube. The resulting solution was stored on ice and gently shaken every 10 min. After 30 min, the tube was sonicated for 10–15 s, followed by centrifugation at 14 000 rpm for 10 min. The supernatant was used for SERS characterization.

##### Antibody and Protein Immobilization on the Substrate

All substrates were prepared by cutting cover glass to a size of 0.5 × 0.5 cm and cleaned using a piranha solution (H_2_SO_4_:H_2_O_2_ = 3:1; caution: extremely harmful to the human body). To conjugate BSA on the APTES‐functionalized substrate, the cleaned substrate was immersed in APTES (1% v/v) for 2 h and then thoroughly rinsed with deionized water (DIW). The functionalized substrate was immersed in BSA (1% w/v) that was diluted in PBS for 8 h.

For the SERSIA, antibody solution (8 µg mL^−1^) in the PBS was mixed with APTES (1% v/v) at a ratio of 1:1. The cleaned substrate was then incubated in the mixed antibody solution for 30 min. Following washing with PBS, the antibody‐immobilized substrate was blocked with BSA (1% w/v) for 30 min at 37 °C, followed by washing with excessive PBS. Subsequently, the biological sample that contained antigens was treated with the antibody‐immobilized substrate for 1 h at 37 °C. Following the immobilization of the antigens, the substrate was thoroughly washed with excessive PBS.

For the control case in which the antigens were attached to the antibody‐immobilized GNPs, the cleaned substrate was immersed in APTES (1% v/v) for 1 h and then washed. In addition, the GNP colloidal solution was coated on the substrate using our centrifugation method. After the substrate was washed with DIW, the surface of the GNPs was modified through incubation in MPA (10 mm) in ethanol for 12 h. After washing, EDC and NHS (both 0.2 m in PBS) were mixed at a ratio of 1:1 v/v. Next, the MPA‐coated GNP substrate was incubated in the mixed EDC and NHS solution (both 0.2 m in PBS) at a ratio of 1:1 for 1 h, followed by washing with PBS. Then, the antibody (100 µg mL^−1^) in PBS was treated with the substrate for 1 h and washed. The resulting substrate was blocked with BSA (1% w/v) for 20 min and washed. Then, the antigen solution was added, and the substrate was incubated for 1 h.

##### GNP Coating by Centrifugation

GNP colloidal solution was added to the centrifugal tube in which the substrate was placed. The tube was centrifuged at 1000 × *g* for 3 min. The supernatant was removed gently through a pipette. For additional accumulation of GNPs, the procedure for adding GNPs and removing the supernatant was repeated. The final GNP‐coated substrate was rinsed with DIW and dried using pure N_2_ gas.

##### SERS Characterization

All SERS measurements were performed using an inverted‐type microscope (Axio Observer D1) purchased from Zeiss. The microscope was equipped with a spectrometer (Acton SP2300) from Princeton Instruments. The SERSIA substrate was irradiated with a 1.5‐mW 785‐nm laser, and then scattered light from the substrate passed through the 785 nm filter. A cooled spectrograph detector (PIXIS400, Princeton Instruments) with a resolution of 1340 × 400 pixels was used to scan the Raman spectra. For protein quantification, the acquisition time was 10 s. For protein imaging, the acquisition region was 2 × 2 µm, and the acquisition time was 5 s. The spectral signals were adjusted and denoised using chromatogram baseline estimation and denoising using the sparsity (BEADS) method.^[^ ^22^ ^]^ All numerical calculations, including the preprocessing of the spectral data and similarity analysis, were performed using MATLAB R2017a.

## Conflict of Interest

The authors declare no conflict of interest.

## Supporting information

Supporting Information

## References

[1] a) B. Bouvier , L. J. Blum , Anal. Lett. 2005, 38, 1491;

[2] a) H. Shin , H. Jeong , J. Park , S. Hong , Y. Choi , ACS Sens. 2018, 3, 2637; 30381940 10.1021/acssensors.8b01047

[3] a) S. Fang , Z. Wang , Drug Des., Dev. Ther. 2014, 8, 1595; 10.2147/DDDT.S69690 PMC4189714 25302015

[4] I. Bruzas , W. Lum , Z. Gorunmez , L. Sagle , Analyst 2018, 143, 3990. 30059080 10.1039/c8an00606g

[5] a) S. Hong , O. Shim , H. Kwon , Y. Choi , Anal. Chem. 2016, 88, 7633; 27396542 10.1021/acs.analchem.6b01451

[6] N. Feliu , M. Hassan , E. Garcia Rico , D. Cui , W. Parak , R. Alvarez‐Puebla , Langmuir 2017, 33, 9711. 28826207 10.1021/acs.langmuir.7b01567

[7] T. Demeritte , R. Kanchanapally , Z. Fan , A. K. Singh , D. Senapati , M. Dubey , E. Zakar , P. C. Ray , Analyst 2012, 137, 5041. 22970432 10.1039/c2an35984g

[8] Z. Wang , S. Zong , L. Wu , D. Zhu , Y. Cui , Chem. Rev. 2017, 117, 7910. 28534612 10.1021/acs.chemrev.7b00027

[9] a) A. San Paulo , R. Garcia , Biophys. J. 2000, 78, 1599; 10692344 10.1016/S0006-3495(00)76712-9 PMC1300757

[10] L.‐J. Xu , C. Zong , X.‐S. Zheng , P. Hu , J.‐M. Feng , B. Ren , Anal. Chem. 2014, 86, 2238. 24460183 10.1021/ac403974n

[11] L. Litti , M. Meneghetti , Phys. Chem. Chem. Phys. 2019, 21, 15515. 31259983 10.1039/c9cp02015b

[12] J. Schwan , S. Ulrich , V. Batori , H. Ehrhardt , S. Silva , J. Appl. Phys. 1996, 80, 440.

[13] Y. Sun , M. Yanagisawa , M. Kunimoto , M. Nakamura , T. Homma , Spectrochim. Acta, Part A 2017, 184, 1. 10.1016/j.saa.2017.04.036 28475958

[14] a) I. H. Boyaci , H. T. Temiz , H. E. Geniş , E. A. Soykut , N. N. Yazgan , B. Güven , R. S. Uysal , A. G. Bozkurt , K. İlaslan , O. Torun , RSC Adv. 2015, 5, 56606;

[15] P. Wang , X. Wang , L. Wang , X. Hou , W. Liu , C. Chen , Sci. Technol. Adv. Mater. 2015, 16, 034610. 27877797 10.1088/1468-6996/16/3/034610 PMC5099834

[16] S. K. Vashist , E. M. Schneider , E. Lam , S. Hrapovic , J. H. Luong , Sci. Rep. 2015, 4, 4407. 10.1038/srep04407 PMC3957147 24638258

[17] M. Braiek , K. Rokbani , A. Chrouda , B. Mrabet , A. Bakhrouf , A. Maaref , N. Jaffrezic‐Renault , Biosensors 2012, 2, 417. 25586032 10.3390/bios2040417 PMC4263564

[18] Z. Andreu , M. Yáñez‐Mó , Front. Immunol. 2014, 5, 442. 25278937 10.3389/fimmu.2014.00442 PMC4165315

[19] J. Wang , W. Chen , W. Wei , J. Lou , Oncotarget 2017, 8, 96215. 29221200 10.18632/oncotarget.21894 PMC5707094

[20] R. Kengne‐Momo , P. Daniel , F. Lagarde , Y. L. Jeyachandran , J.‐F. Pilard , M. J. Durand , G. Thouand , Int. J. Spectrosc. 2012, 2012, 1.

[21] Z. Xu , M. Xia , Inf. Sci. 2011, 181, 2128.

[22] X. Ning , I. W. Selesnick , L. Duval , Chemom. Intell. Lab. Syst. 2014, 139, 156.

