# Supplementary material for: Protein Quantification and Imaging by Surface‐Enhanced Raman Spectroscopy and Similarity Analysis
Source: Adv Sci (Weinh). 2020 Apr 16;7(11):1903638. doi: 10.1002/advs.201903638 (PMC7284192; doi:10.1002/advs.201903638)
Supplement: Supplementary file 1 — Supporting Information [file ADVS-7-1903638-s001.pdf]

Copyright WILEY-VCH Verlag GmbH & Co. KGaA, 69469 Weinheim, Germany, 2020.

# Supporting Information

**Protein quantification and imaging by surface-enhanced Raman spectroscopy and similarity analysis**

*Hyunku Shin, Seunghyun Oh, Daehyeon Kang, and Yeonho Choi**

## Calculation of the enhancement factor (EF)

We calculated the SERS substrate enhancement factors (EFs) using the following equation:

$$\mathrm{EF} = \frac{I_{SERS}/N_{Surf}}{I_{RS}/N_{Vol}}$$

where $I_{SERS}$ is the SERS intensity of 4-ATP at around 1085 $cm^{-1}$ (the characteristic Raman band of 4-ATP), $I_{RS}$ is the Raman scattering intensity of 4-ATP powder, $N_{Surf}$ is the number of molecules on the surface of the SERS substrate, and $N_{Vol}$ is the number of molecules in the focused laser volume. As shown in figure S3, the $I_{SERS}$ was 805. To determine the $I_{RS}$, the powder of 4-ATP was observed with the same optical setup of the SERS measurement, showed the intensity of 8. To calculate the number of 4-ATP molecules, the laser focused spot was almost 1 μm and the penetration depth (as the Raman scattering-effective height) was set to be 16.5 μm. Thus, the laser-focused volume was calculated by approximately 13.0 $\mu m^3$. Using the density (1.18 $g/cm^3$) and molecular weight (125.19 g/mol) of the solid 4-ATP, the $N_{Vol}$ was calculated to be 7.36 x $10^{10}$ molecules in the focused volume.[1] To determine the $N_{Surf}$ , we first measured the number of nanoparticles in the focused area. Through SEM images, the number of particles was measured to be 29 particles in the focused area, on average. To estimate the number of the adsorbed molecules on the surface of particles, we assumed that the 4-ATP molecules fully cover the whole surface of particles. Thus, we determined the $N_{Surf}$ of 3.62 x $10^5$ molecules.

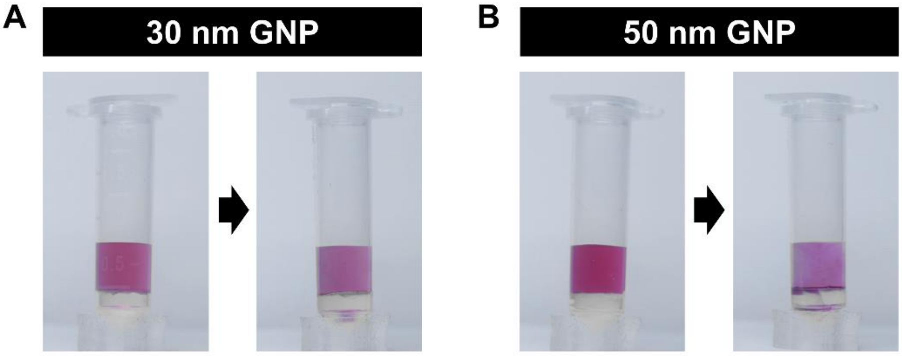


**Figure S1.** Size-dependent sedimentation of GNPs by centrifugation. The sedimentation of (A) 30-nm and (B) 50-nm GNPs was not significant after centrifugation at 1,000 × g for 3 min.

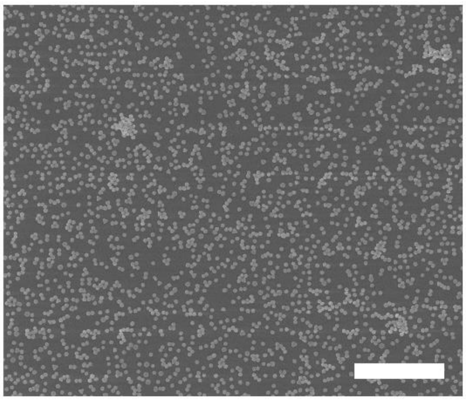

**Figure S2.** SEM image of coated GNPs on an APTES-functionalized cover glass surface. The scale bars indicate 2 µm.

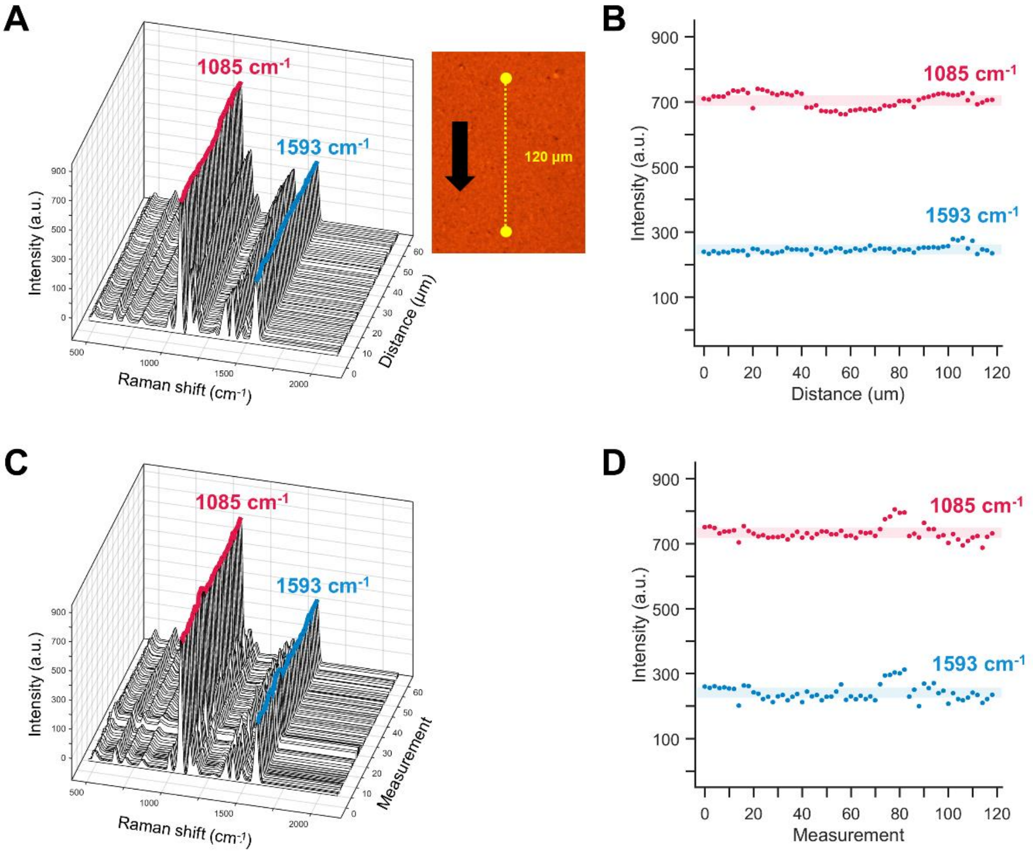


**Figure S3.** Signal uniformity of the SERS substrate. (A, C) SERS spectra of 4-ATP at 2-μm intervals and random measurement spots, respectively. (B, D) Tendencies of the SERS intensities at the characteristic Raman bands of 4-ATP.

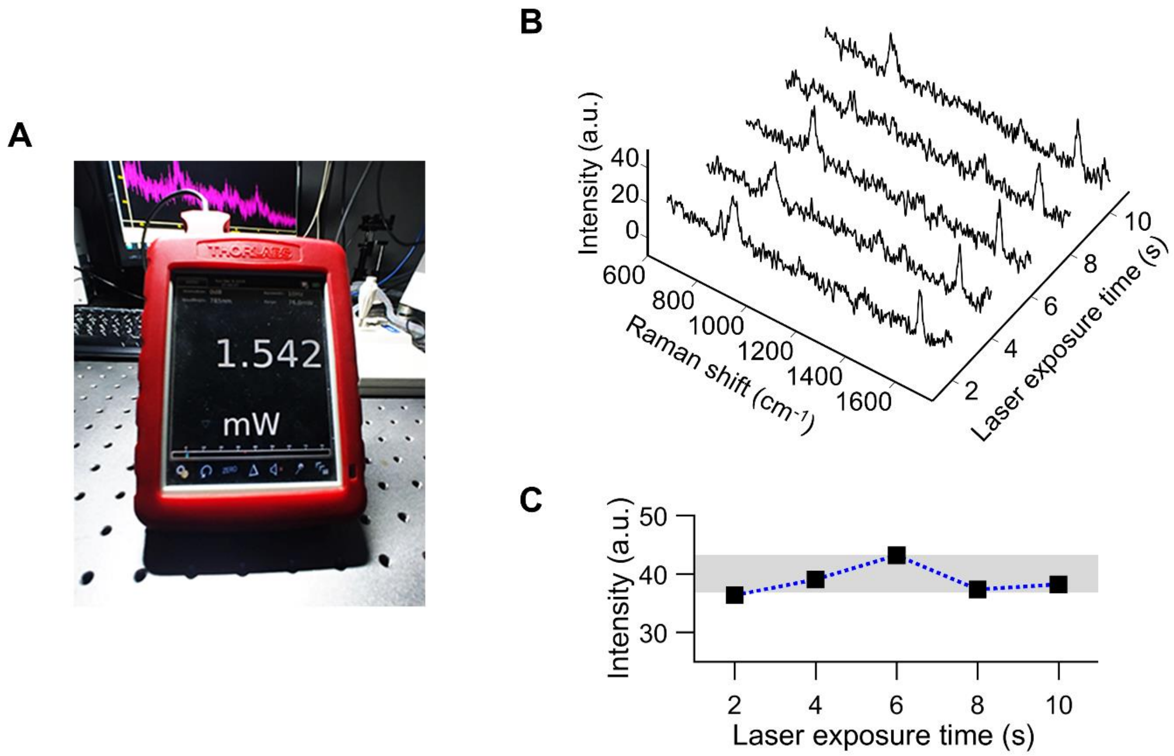


**Figure S4.** Photothermal stability test. (A) Measured laser power. (B) SERS signals of a BSA-conjugated substrate at intervals of 2 s. (C) Fluctuation in intensity at a 1620-cm$^{-1}$ Raman shift. The gray square indicates the noise range

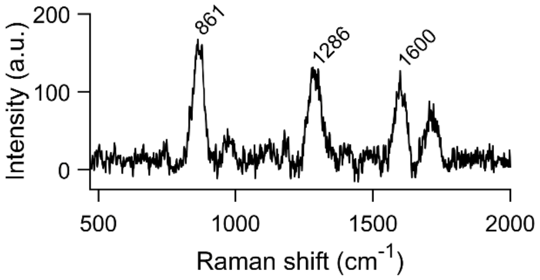


**Figure S5. SERS signal of citrate on GNPs.** The signal was obtained from dried aggregates of citrate-capped GNPs.

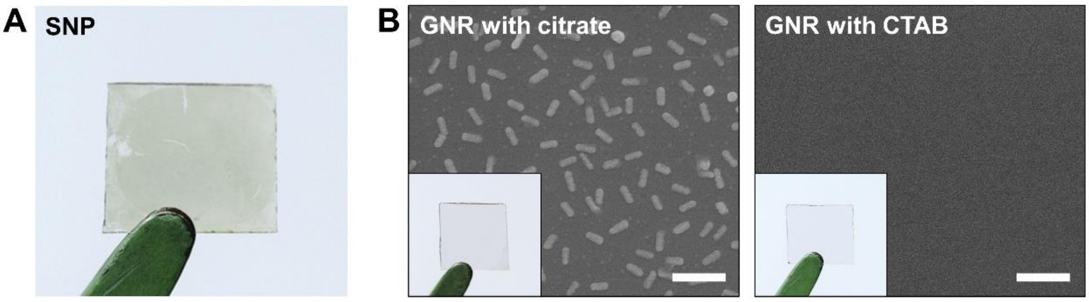


**Figure S6.** Centrifugation-based coating of other plasmonic nanoparticles. (A) Photograph of an SNP-coated substrate. (B) Photographs and SEM images of GNRs with citrate and CTAB. The citrate and CTAB provide negatively and positively charged surfaces for the nanorods, respectively. The scale bars indicate 200 nm.

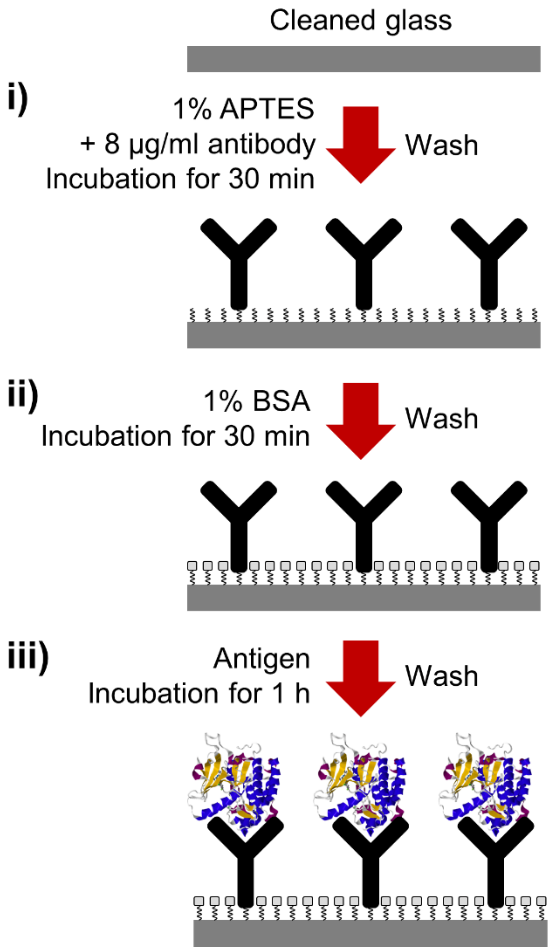


**Figure S7.** Preparation of the SERSIA substrate: i) antibody immobilization; ii) blocking with BSA; iii) conjugation of antigens.

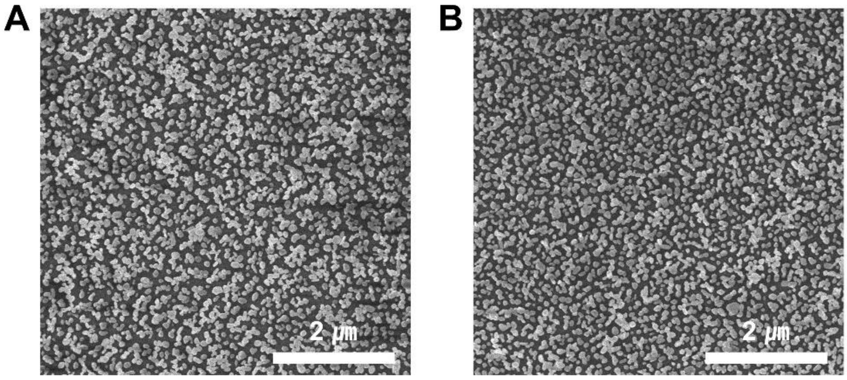


**Figure S8.** Coverage of GNPs on the substrate. SEM images of the (A) antibody-only and (B) antigen-conjugated samples.

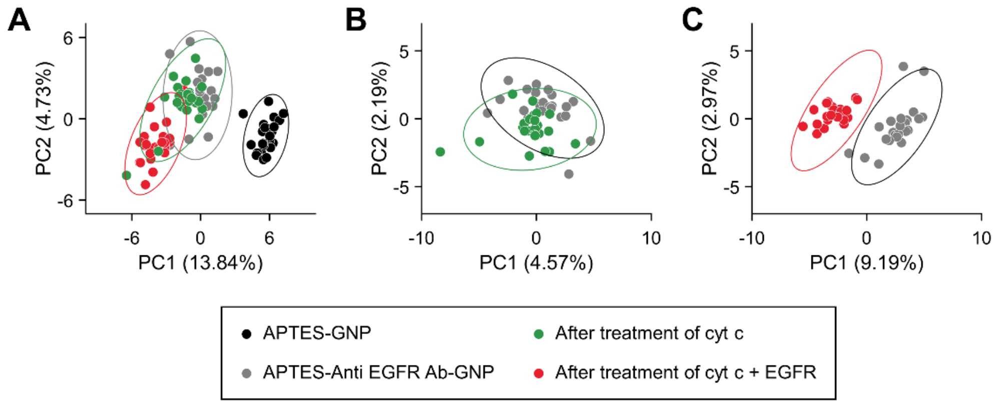


**Figure S9.** Off-target test. Dots represent: (black) APTES-GNP, (grey) APTES-Anti EGFR Ab-GNP, (green) off-target sample, and (red) on-target sample. The off-target sample contains cytochrome c, excluding the target protein, EGFR. The on-target sample contains both cytochrome c and EGFR.

**Reference**

[1] Y. Wang, H. Chen, S. Dong, E. Wang, *J. Chem. Phys.* **2006**, *124*, 074709.
